# Lower Expression of TLR2 and SOCS-3 Is Associated with *Schistosoma haematobium* Infection and with Lower Risk for Allergic Reactivity in Children Living in a Rural Area in Ghana

**DOI:** 10.1371/journal.pntd.0000227

**Published:** 2008-04-16

**Authors:** Franca C. Hartgers, Benedicta B. Obeng, Yvonne C. M. Kruize, Marjolijn Duijvestein, Anna de Breij, Abena Amoah, Irene A. Larbi, Ronald van Ree, Michael D. Wilson, Laura C. Rodrigues, Daniel A. Boakye, Maria Yazdanbakhsh

**Affiliations:** 1 Department of Parasitology, Leiden University Medical Centre, Leiden, The Netherlands; 2 Noguchi Memorial Institute for Medical Research, University of Ghana, Legon, Accra, Ghana; 3 Department of Experimental Immunology, Academic Medical Center, Amsterdam, The Netherlands; 4 Department of Infectious Diseases, London School of Hygiene & Tropical Medicine, London, United Kingdom; University of Edinburgh, United Kingdom

## Abstract

**Background:**

Helminth infections are prevalent in rural areas of developing countries and have in some studies been negatively associated with allergic disorders and atopy. In this context little is known of the molecular mechanisms of modulation involved. We have characterized the innate immune responses, at the molecular level, in children according to their helminth infection status and their atopic reactivity to allergens.

**Methodology/Principal Findings:**

The mRNA expression of several genes of the innate immune system that have been associated with microbial exposure and allergy was examined in 120 school children in a rural area in Ghana. Helminth infections were common and atopy rare in the study area. The analysis of gene expression in ex vivo whole blood samples reflected the levels of corresponding proteins. Using this approach in a population of school children in whom the presence of *Schistosoma haematobium* infection was associated with protection from atopic reactivity, we found that the level of TLR2 and SOCS-3, genes associated with atopy in the children, were significantly downregulated by presence of *S. haematobium* infection.

**Conclusions:**

*S. haematobium* infections modulate the expression of genes of the innate immune system (TLR2 and SOCS-3); these are genes that are associated with increased allergic inflammatory processes, providing a molecular link between the negative association of this infection and atopy in rural children in Ghana.

## Author Summary

Inflammatory diseases such as atopic disorders are a major health problem in the Western world, but their prevalence is also increasing in developing countries, especially in urban centres. There is increasing evidence that exposure to a rural environment with high burden of compounds derived from parasites and microorganisms is associated with protection from atopic disorders. Since urbanisation is progressing at a rapid pace, particularly in less-developed nations, there is a need to understand the molecular processes that control the progress towards the development of allergic diseases in developing countries. In this study we have examined a population of school children living in a rural area of Ghana, where helminth (worm) infections are prevalent and associated with protection from skin reactivity to house dust mite. Blood samples were collected from these children and analysed for the expression levels of several genes involved in the development of a pro allergic immune system. The results point at a potential molecular link that might explain the negative association between schistosome infections and allergies.

## Introduction

In the last few decades, allergic diseases have become a major health burden in the western world. Although these disorders clearly have a genetic component, their rapid change in prevalence points to environmental conditions that have changed during this time frame. In the same time frame, there has been a decrease in exposure to microbial products as a result of changing lifestyle with, among others, improved sanitation and access to clean water. Interestingly, in the developing world, the prevalence of allergies is relatively low, particularly in rural areas, where exposure to infectious agents is high. There is increasing evidence that exposure to pathogen-derived compounds influences the maturation of the immune system and therefore the balance reached between pro- and anti-inflammatory responses, such that Th2 responses are kept under control when allergens are encountered.

In rural areas in the developing world, chronic helminth infections are highly prevalent. These infections not only result in skewing of the immune responses towards Th2, but also induce the higher production of anti-inflammatory molecules such as IL-10 to prevent the elimination of helminths, which at the same time protect the host against the pathological consequences of excessive inflammation [1]. Such an anti-inflammatory environment induced by chronic helminth infections might modulate immune responses to other antigens. For example, chronic infection with schistosomes or *Onchocerca* was shown to modulate the immune response to tetanus toxoid following vaccination [2],[3].

Epidemiological studies have revealed both positive and negative associations between helminth infections and allergies (reviewed in [4]). It is thought that severe, chronic infections are often associated with suppression of allergic reactivity. For example chronic infections with intestinal helminth, such as with hookworm, have been shown to suppress allergic diseases [5],[6]. These observations have been confirmed for schistosomiasis, demonstrating lower skin reactivity to allergen in infected individuals [7],[8]. Additionally, removal of helminths by long-term anti-helminth treatment in Venezuelan or Gabonese children resulted in increased atopic reactivity to house dust mite [9],[10], even though a shorter anti-helminth treatment did not show an effect on atopy in one study [11].

In a population of rural Ghanaian school children a negative association was found between infection with *Schistosoma haematobium* and skin reactivity to mite allergen (Obeng et al, submitted for publication). Within this study we aimed to identify the molecular mechanisms by which schistosome infections may modify immune responses and modulate inflammatory reactions such as atopy. To address this we selected two groups of genes that have been described previously to play a role in allergic diseases. Toll-like receptors (TLRs) have been shown in several studies outside Africa to change in expression levels following exposure to microorganisms [12]–[15]. In a European study these molecules were linked to allergy: children of farmers in Alpine regions, exposed to high microbial burden and with a low prevalence of atopy, had altered levels of TLR2 [16], suggesting that exposure to microorganisms might modulate the innate immune system and thereby suppress the development of allergic disorders. The molecules suppressor of cytokine signalling (SOCS)-1 and SOCS-3 have recently been described and reported to be involved in TLR signalling and inflammatory diseases [17]–[22]. Elegant studies by Kubo and co workers in animal models have shown SOCS-3 to be involved in regulation of immune responses in allergic disease [23],[24].

Given that helminth products have been shown to modulate cells of the innate immune system and to interfere with pathways that are activated via TLR stimulation [15],[25], we asked whether in an area in Africa where helminth infections are highly prevalent and allergic disorders are low, we can find molecular pathways that may explain the relationship. To this end, gene expression not only of TLRs but also of molecules such as SOCS-1 and SOCS-3, involved in downstream signalling, were studied in whole blood samples of rural Ghanaian school children.

The results of this study showed that high expression of TLR2 and SOCS-3 was associated with allergic skin reactivity, whereas helminth infection was associated with lower expression levels of TLR2 and SOCS-3, providing a potential regulatory link between helminth infection and allergies at the molecular level.

## Methods

### Study population

The study population consisted of schoolchildren between 5 and 14 years of age. Children whose parents consented by signing or thumb printing an informed consent form were registered to participate in a large study on allergy and parasitic infections (B.B. Obeng *et al*, manuscript submitted). The Institutional Review Board of the Noguchi Memorial Institute for Medical Research, Accra, Ghana approved the study. Skin reactivity to mite was negatively associated with *S. haematobium* infection (OR 0.5, 95% CI 0.2–1.0, p = 0.05), particularly in areas where prevalence of schistosomiasis is high (OR 0.3, 5% CI 0.1–0.9, p = 0.04). Blood samples from children from two rural schools with high prevalence of *S. haematobium* infection were used for RNA isolation to study gene expression. In these schools, the reactivity to house dust mite was low (9%) compared to school children from Accra (15%), free of any helminth infections, and with a relatively high socioeconomic status.

The study subjects were fist selected randomly, one out of three children from whom blood samples were available were selected (107 children). In order to increase power, all skin prick test (SPT) positive children from these schools were added to our randomly selected subjects, along with randomly selected SPT negative children (16 children in total), resulting in a group of 123 children (Table 1).

**Table 1:** Characteristics of study population.

	School children n = 123
**Age (yrs)**	
median	9.0
(min-max)	(5–14)
**Sex**	
M/F (% M)	66/57 (54%)
**Helminth infection**	
at least one helminth (%)	60/113 (52%)
*S. haematobium* (%)	46/120 (38%)
hookworm (%)	29/119 (24%)
other helminth (%)	4/120 (3%)
**Schistosome egg load**	
GM (eggs/10 ml urine)	35
(min-max)	(1–1112)
**Hookworm egg load**	
GM (eggs/gram faeces)	186
(min-max)	(20–3540)
**Malaria infection (%)**	59/117 (50%)

Egg loads are represented as geometric means for infected individuals only

Malaria infection was examined by microscopic examination of thick blood smears

### Detection of helminth and malarial infection

The participants were given specimen bottles and were asked to collect a fresh stool and urine sample for the detection of helminth infections. Stool examination was performed by the Kato-Katz method for the detection of hookworm and trichuris, and the total number of eggs was calculated per gram of faeces. Urine samples were used for the detection of *S. haematobium* by passing 10 ml of urine through a filter with 10-micron pore size. A subject was considered positive for helminth infection if eggs of any of the helminth species were detected.

Blood samples were collected from all participants for the detection of the malaria infection by Giemsa-stained thick smear (GTS) examination.

### Skin prick test for mite allergen

The immediate hypersensitivity skin prick test with inhalant allergen extracts was performed by using the standard prick method on the volar surface of the right forearm with the standardized extracts of 6 allergens; *Dermatophagoides pteronyssinus* (Der P), *Dermatophagoides farinae* (Der F), cat, dog, peanut and grass mix (HAL Allergen Laboratories, The Netherlands). Histamine dihydrochloride (1/1000) and glycerinated saline solution were used as positive and negative controls, respectively. The wheal diameter was measured after 15 minutes and the result considered positive when the wheal size was at least 3 mm in diameter, in the absence of significant reactivity of the diluent negative control. None of the children were taking anti-allergic medicine that might interfere with SPT or had ever been treated with specific immunotherapy. Since most of the positive reactions seen were against mite allergen, we have focused on children having a positive skin test for mite allergen.

### Detection of total and mite-specific IgE

Serum levels of total IgE were measured by the enzyme linked immunosorbent assay (ELISA) as described before [26]. Results were expressed as international units per ml (IU/ml).

Serum levels of house dust mite (HDM) antibodies were determined by radio allergosorbent test (RAST) as described previously [27] (CLB, Amsterdam, The Netherlands). Results were expressed as international units per ml (IU/ml). One IU is 2.4 ng IgE. Subjects were considered sensitised when concentrations of specific IgE of more than 0.7 IU/ml were measured.

### RNA isolation from whole blood

Immediately after venapuncture into heparinised tubes, 0.8 ml of whole blood was added to 3.6 ml of Nuclisens lysis buffer (Biomérieux, Boxtel, The Netherlands) to stabilise the RNA. Samples were stored for a maximum of two weeks at 4°C, after which they were put at −80°C for long-term storage. The Nuclisens Isolation kit (Biomérieux) was used for the isolation of total nucleic acid (approximately 1 ml of blood mixed with lysis buffer per isolation) according to the manufacturer's instructions. Genomic DNA was removed by treating the samples with RNAse-free DNAse (Invitrogen, Breda, The Netherlands) for 30 minutes at 37°C, followed by the Nuclisens isolation procedure to isolate the purified RNA. RNA was isolated from the same samples before and after one year of storage at −80°C, and mRNA levels of several genes of interest were compared. There was no difference in gene expression indicating that mRNA was stable in this buffer for at least one year at −80°C, and that the procedure was consistent.

### RNA isolation from cell subsets

From a subset of the donors, PBMC were isolated and monocytes and T cells were separated by subsequent labelling and magnetic cell separation of cells with CD3 and CD14 Microbeads (Miltenyi Biotech, Germany). Fractionated monocytes and T cells were mixed with Nuclisens lysis buffer. Fluorescence activated cell sorting (FACS) of the isolated cells indicated that these fractions were at least 90% pure. The unlabeled cell fraction depleted for monocytes and T cells was also collected and mixed with lysisbuffer (remaining cell fraction). Samples were stored and RNA was isolated as described for whole blood samples. The percentages of monocytes and T cells in all donors were determined by flow cytometry of the PBMC prior to the isolation procedure.

### cDNA synthesis and real-time PCR

Reverse transcription of RNA was performed using moloney murine leukaemia virus reverse transcriptase (M-MLV RT) (Invitrogen). Samples without RT were regularly taken along to control for genomic DNA contamination. Gene expression was assessed with real-time quantitative PCR (Prism 7700, Applied Biosytems). PCR reactions were performed in duplicate in accordance with the Taqman^TM^ assay instructions using Taqman probes and qPCR Core kit reagents (both Eurogentec, Seraing, Belgium). Gene expression was normalized to the housekeeping gene 18S rRNA and calculations were performed as described [28]. Analysis of the expression of 8 different housekeeping genes in a subset of the samples indicated that 18S rRNA was a stable housekeeping gene in our samples. Sequences of primers and probes have been obtained from Dr. Roger Lauener (TLR2, TLR4, 18S rRNA, [16]) and from Dr. Masato Kubo (SOCS-1, SOCS-3, [23]). IgE mRNA levels were determined by a primer and probe set specific for the CH1 region of IgE (forward primer 5′-CAA TGCCACCTCCGTGACTC-3′, reverse primer 5′-CGTCGCAGGACGACTGTAAG-3′ and probe 5′-ATCGTCCACAGACTGGGTCGACAACAAA-3′). For each gene, after normalisation for the housekeeping gene, the donor with the lowest expression was set to 1. For the expression levels of TLR2 and SOCS-3 in isolated cell subsets, expression of SOCS-3 or TLR2 in T cells was set to 1 for each donor.

### Comparison of mRNA and surface expression of TLR2

Whole blood from 5 donors was diluted 1∶1 with RMPI 1640 medium (Gibco) and stimulated for 16 hours and 24 hours in 96-well round bottom plates with medium, 10 µg/ml SEA (schistosomal egg antigens), 100 ng/ml LPS (Sigma), 100 µg/ml poly I:C or 5 µg/ml TNF-α (Sanquin, The Netherlands). After 16 hours the blood was mixed with ABI Lysis buffer (Applied Biosystems) and RNA was extracted using the ABI6100 according to their protocol (Applied Biosystems). cDNA synthesis and quantitative PCR for TLR2 was performed as described above. 24 hours after stimulation, cells were mixed with FACS lysing solution (BD Biosciences) to lyse the erythrocytes, washed with PBS and cells stained with anti-TLR2 PE (clone T2.5, eBioscience). Flow cytometric analyses were performed using a Becton Dickinson FACSCalibur flow cytometer (BD Biosciences) and analysed using FlowJo analysis software (Tree Star Inc.).

### Statistical analysis

Association of total and mite-specific IgE with skin reactivity to mite was analysed by logistic regression of log-transformed values. The association of gene expression with skin reactivity or helminth infection was analysed by logistic regression using a value of each gene to separate high and low gene expression, since the association between gene expression and allergen reactivity might not be a linear one. This value was based on the geomean of the relative expression data (low expression: below geomean; high expression: above geomean). The regression analysis was performed adjusting for age, sex and helminth infection (Table 2) or for age, sex and skin reactivity to mite (Table 3). The comparison of the non-adjusted means of gene expression between skin prick positive and negative children and between helminth-infected and non-infected children was determined by the non-parametric Mann-Whitney test. Correlation between mRNA and surface TLR2 levels and between mRNA and serum IgE levels was compared using the non-parametric Spearman's correlation test.

**Table 2:** Determinants of a positive skin prick reactivity to house dust mite.

	Adjusted OR [95% CI]*	P
**total IgE** $	1.93 [0.78–4.75]	0.16
**mite IgE** $	9.90 [2.57–38.26]	0.001
**TLR2** #	2.56 [0.79–8.29]	0.12
**TLR4** #	0.93 [0,32–2,74]	0.90
**SOCS-1** #	5.85 [1.52–22.42]	0.01
**SOCS-3** #	4.44 [1.27–15.53]	0.02

*: adjusted for age, sex and helminth infection

$ log-transformed

# high expression of indicated genes defined as levels above the geomean of all samples tested (3.2 for TLR2; 5.0 for TLR4;3.6 for SOCS-1; 6.0 for SOCS-3)

**Table 3:** Association between helminth infection and gene expression.

	TLR2#		TLR4#		SOCS-1#		SOCS-3#	
	OR*	P	OR*	P	OR*	P	OR*	P
**Helminth**	0.26	0.001	0,40	0.024	1.38	0.42	0.29	0.003
	[0.11–0.60]		[0.18–0,89]		[0.63–3.01]		[0.13–0.66]	
**Schistosomes**	0.24	0.001	0.56	0.15	1,12	0.77	0.35	0.010
	[0.11–0.55]		[0.26–1.24]		[0.51–2.46]		[0.16–0.78]	

*: adjusted for age, sex, and skin reactivity to mite

# high expression of indicated genes defined as levels above the geomean of all samples tested (3.2 for TLR2; 5.0 for TLR4;3.6 for SOCS-1; 6.0 for SOCS-3)

## Results

### Characteristics of the study population in rural Ghana

The study population here originates from two schools selected from a large study on allergy and parasitic infections. The schools were in a rural area highly endemic for helminth infections (see Material and Methods section). Fifty-four percent of the children were infected with at least one helminth species (Table 1). As indicated in Table 1, the most prevalent helminth species was *Schistosoma haematobium*, followed by hookworm, *Ascaris lumbricoides* and *Trichuris trichiura*. In the population where mRNA expression was analyzed (see Materials and Methods), 14 out of 74 schistosome negative children had a positive skin reaction to mite (19%), whereas 5 out of 46 schistosome-infected children were SPT positive for mite (11%) resulting in a significant negative association between infection with *S. haematobium* and atopy (OR 0.26 [0.07–1.00], p = 0.05, adjusted for age, sex, school and levels of mite IgE). Additionally, the odds ratio of the association between the log of mite IgE and atopy is clearly lower in children infected with helminths (OR 5.8 [1.0–33.2]; p = 0.05) compared to the odds ratio in non-infected children (OR 18.7 [2.4–145.8]; p = 0.005).

The level of mite-specific IgE was a strong determinant for the risk of positive skin reactivity to mite (Table 2). In contrast, the level of total IgE was not significantly associated with atopy. However, total IgE was associated with atopy in the children that were not infected with helminths (OR = 5.6, CI 95%: 1.0–30.9; p<0.05).

### Validation of ex vivo mRNA expression profiles

**Figure 1:**
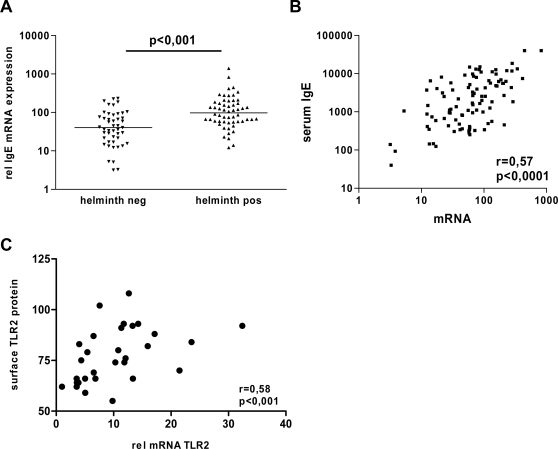
Validation of *ex vivo* mRNA expression profiles. (A) Relative IgE mRNA expression in blood of helminth-infected (helminth pos) and non-infected (helminth neg) individuals. Horizontal bars represent median values per group. (B) Scatter plot showing correlation between levels of IgE mRNA and serum levels of IgE. (C) Scatter plot showing correlation between TLR2 mRNA expression in whole blood measured by real-time PCR (16 hours after stimulation) and surface expression of TLR2 by flow cytometry in same samples (24 hours after stimulation).

In order to evaluate whether the *in vivo* status of the immune system could be evaluated by analysing the mRNA expression in whole blood, RNA was isolated from peripheral blood samples collected in our study population. In agreement with IgE serum levels, the levels of IgE mRNA were significantly higher in helminth-infected children compared to non-infected children (Figure 1A), and a high correlation was seen between the mRNA expression and serum IgE levels (r = 0.58, p<0.001; Figure 1B). In addition the mRNA levels of TLR2 were compared to surface TLR2 protein expression using flow cytometry in whole blood samples. There was a strong correlation between TLR2 mRNA expression and TLR2 surface expression (r = 0.58; p<0.001), validating the use of mRNA levels measured in whole blood samples as a reflection of the measured immunological events *in vivo*.

### The association of TLR2, SOCS-1 and SOCS-3 mRNA expression and skin reactivity to mite allergen

**Figure 2:**
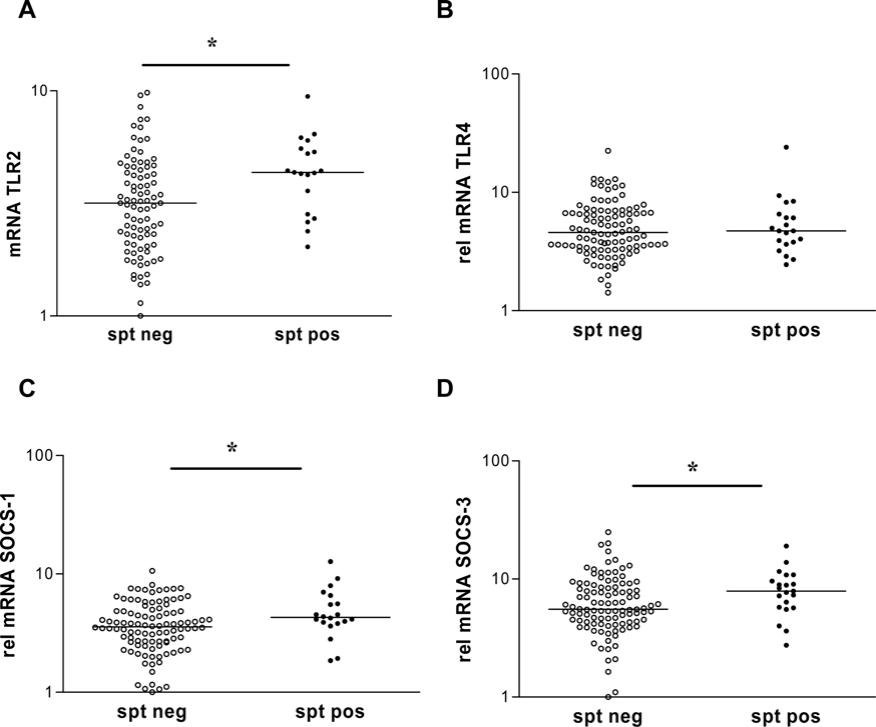
Relative gene expression levels of TLR2, TLR4, SOCS-1 and SOCS-3 in skin test positive and negative children. Relative mRNA expression levels of TLR2 (A), TLR4 (B), SOCS-1 (C) and SOCS-3 (D) in blood of children positive (spt pos) or negative (spt neg) for skin reactivity to house dust mite. Horizontal bars represent median values per group. * p<0.05.

In Ghanaian school children living in an area highly endemic for parasitic infections, there was a significantly higher expression of TLR2 in subjects with positive skin reactivity to house dust mite (Figure 2A); high expression of TLR2 doubled the risk of atopy (OR 2.6, Table 2), whereas there was no such association for TLR4 and skin reactivity (OR 0.9; Table 2 and Figure 2B). Children with high expression of SOCS-1 or SOCS-3 had a significantly increased risk for skin reactivity (OR 5.8 and 4.4, respectively, Table 2). Both SOCS-1 and SOCS-3 expression were significantly elevated in those who were skin prick positive to house dust mite compared to non-atopic children (Figures 2C and 2D).

**Figure 3:**
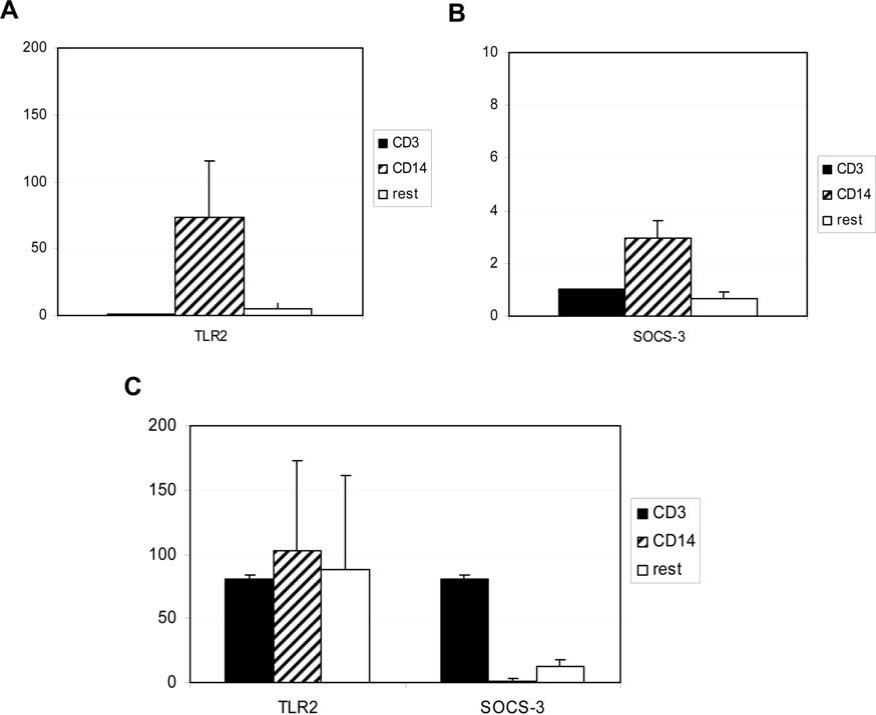
Expression of TLR2 and SOCS-3 mRNA in cell subsets. Mean of relative mRNA expression of TLR2 (A) and SOCS-3 (B) in T cells (CD3), monocytes (CD14) and cells depleted of T cells and monocytes (rest) from 5 donors. Y error bars indicate the standard error of the mean from each group. (C) Mean expression levels of TLR2 and SOCS-3 in T cells, monocytes and the rest of the cells corrected for the percentages of cells within the PBMC population for each donor.

To determine the source of TLR2 and SOCS-3 expression in whole blood samples, monocytes and T cells were isolated from five donors. Although the mRNA expression of TLR2 was high in monocytes (63 to 275-fold higher than in T cells, Figure 3A), correction for the percentages of monocytes and T cells in the peripheral blood mononuclear cells, indicated that monocytes, T cells and other cells contributed similarly to the TLR2 expression measured in whole blood (Figure 3C). In contrast, the mRNA expression of SOCS-3 could clearly be attributed to the T cell fraction with little contribution from monocytes or other cells (Figure 3B–C).

### Expression of TLR2 and SOCS-3 mRNA is lower in helminth-infected children

**Figure 4:**
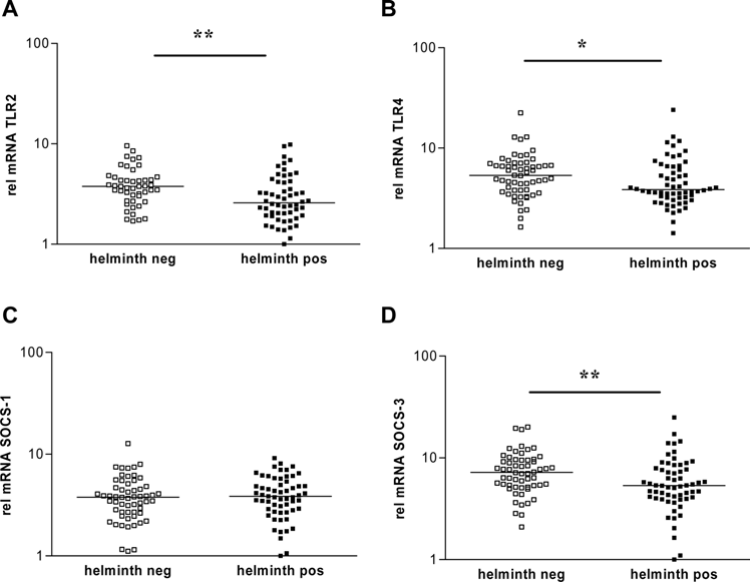
Relative gene expression levels of TLR2, TLR4, SOCS-1 and SOCS-3 in helminth-infected and helminth free children. Relative mRNA expression levels of TLR2 (A), TLR4 (B), SOCS-1 (C) and SOCS-3 (D) in blood of children positive (helminth pos) or negative (helminth neg) for helminth infection. * p<0.05; ** p<0.01.

TLR expression can be altered following exposure to ligands expressed by microorganisms and parasites. Helminth parasites carry signature molecules that can interact with TLRs and therefore could affect their expression. The expression of both TLR2 and TLR4 genes was lower in children with a helminth infection; the effect being more prominent for TLR2 (Figures 4A and 4B). Indeed, infection with helminths predicted low expression of TLR2 and, to a lesser extent, of TLR4 (Table 3). Two major helminth species prevalent in the study area were *Schistosoma haematobium* and hookworm. The mRNA expression of TLR2 in helminth-infected children was significantly lower only in children infected with *S. haematobium,* which was associated with low expression of TLR2 (Table 3), whereas no such association was found for TLR4. Malaria infection was associated with higher levels of TLR2 expression (not shown), and adjustment for malaria infection did not change the association between helminth infection and TLR2 expression. Thus, induction of lower expression of TLR2 by infection with schistosomes seems to be specific.

The analysis of the expression levels of SOCS-1 and SOCS-3 revealed that children infected with helminths had significantly lower expression levels of SOCS-3, but not of SOCS-1 (Figures 4C and 4D). Helminth positivity in a child was associated with low gene expression of SOCS-3, but not of SOCS-1 (Table 3)). As for TLR2, low expression of SOCS-3 was associated with *S. haematobium* rather than hookworm infection.

## Discussion

Using gene expression profiles in whole blood from children living in a rural area in Ghana, we found that high expression of TLR2, SOCS-1 and SOCS-3 mRNA was associated with positive skin reactivity to house dust mite. Presence of *Schistosoma haematobium* infection, reported to decrease the risk of atopy [7] and observed in the current study, affected the expression levels of TLR2 and SOCS-3, which were significantly lower in infected children.

There are few studies that have looked at the association of TLR expression and allergy, and those that have, are all in European populations [29],[30]. Of these, only one study has investigated the levels of TLR expression in an age group comparable to our study. European children living on farms and reported to have a lower risk of developing atopic disorders were shown to have higher expression levels of TLR2 and CD14, compared to non-farmer children [16]. As farmer children would be expected to be exposed to a high burden of environmental microorganisms, the results are in contrast to the lower expression of TLR2 in our helminth infected subjects compared to uninfected Ghanaian school children. Children living in a rural area in Ghana are expected to have exposures that are higher in intensity, and different in nature in terms of the sort of microorganisms and parasites, compared to European farmer children. Moreover, the European study did not examine the TLR2 levels in atopic and non-atopic individuals as we do here, showing that atopy was associated with high expression of TLR2.

Our data support a role for suppression of atopy by current infection with a systemic helminth, *Schistosoma haematobium*. The finding that *S. haematobium* infected children show a lower expression of TLR2 gene, is supported by the results that baseline expression levels of TLR2 protein were also shown to be lower in individuals infected with another systemic helminth infection, the filarial nematode, *Wuchereria bancrofti* [31],[32]. Importantly, lower expression of TLR correlated with a lower expression of co-stimulatory molecules such as CD80 and CD86 and lower production of the inflammatory cytokines IL-6 and TNF-α [32].

Our results also indicated an association of skin reactivity to house dust mite with higher gene expression for both SOCS-1 and SOCS-3. SOCS genes are involved in the pathogenesis of several inflammatory diseases. They are induced upon cytokine signalling or by stimulation of TLR and limit the production of inflammatory cytokines [33]. In murine models, transgenic over expression of SOCS-3 has been shown to mediate and maintain allergic responses [23]. Furthermore, T cell expression of SOCS-3, but not of SOCS-1, was associated with atopic disease in humans and increased with disease severity [23]. These results suggest that SOCS-3 is involved in Th2 skewed responses. However, although helminth infections are clearly associated with Th2 responses, we have found that SOCS-3 expression is decreased in helminth-infected children. Cell subset analysis indicated that T cells were the main source of SOCS-3 mRNA leading us to conclude that in helminth infected children, with strong Th2 responses, SOCS-3 expression is low in T cells. So although both allergic disorders and helminth infections are characterized by Th2 responses, SOCS-3 is associated with allergic disorders, but not with helminth infection. This would suggest that in allergic subjects, with high expression of SOCS-3, Th2 responses are associated with inflammation; whereas in allergic subjects with a helminth infection and consequent low expression of SOCS-3, Th2 cells are not associated with inflammation. Interestingly, a recent report using T cell-specific SOCS-3 conditional knockout mice indicated that in the absence of SOCS-3 expression, the levels of typical Th2 cytokines in peripheral CD4^+^ T cells were either unaffected (IL-5) or only slightly lower (IL-4), whereas following T cell stimulation, the production of the anti-inflammatory cytokines, IL-10 and TGF-β1, were significantly higher. The abolition of SOCS-3 expression in T cells ameliorated ovalbumin-induced airway hyperresponsiveness *in vivo* [34]. Furthermore, CD4+CD25+foxp3 positive regulatory T cells were shown to have low SOCS-3 expression as compared to Th2 cells, indicating that low SOCS-3 expression in T cells is associated with suppressive function [35]. These data raise the possibility that a decrease in SOCS-3 T cell expression by helminth infection might shift the balance towards a modified Th2 response [36] with a more anti-inflammatory function, and thereby might suppress allergic inflammation [1].

As malaria infection was prevalent in our study area, we looked at this infection and found that it had no effect on atopy (multivariate analysis, data not shown) and interestingly found that malaria infection was associated with a higher expression of TLR2 (data not shown), indicating that different pathogens might induce different regulation of TLR expression. Indeed, other protozoa such as *Entamoeba histolytica* and *Trypanosoma cruzi* inhibit immune responses by down-regulating TLR2 expression [37] or signalling via TLR2 [38],[39]. There are numerous studies supporting either up- or downregulation of TLR2 and TLR4, depending on the stimulus and cell type studied [40]. Both an increase and a decrease in TLR2 expression might reflect repeated TLR stimulation, the direction as well as the downstream signaling pathways being dependent on the type of pathogen. Alternatively, the cytokine environment might influence the expression of TLR. Th1 cytokines such as IFN-γ seems to increase expression levels of TLR [41], whereas Th2 cytokines as IL-4 and IL-13, abundantly present in helminth infected individuals, downregulate TLR expression and function [42],[43]. Thus, the exposure of European farmers to Th1 inducing agents might be reflected in higher TLR2 expression, whereas in our subjects the exposure to Th2 inducing agents might lead to low TLR2 expression. The relationship between TLR2 and SOCS-3 expression might not be a direct one. In rural Ghana, helminth infection is associated with low TLR2 as well as low SOCS-3 expression, whereas the expression of TLR2 is high in a European rural area. If TLR2 expression is merely the result of exposure to pathogens, and low SOCS-3 expression is associated with protection from allergy, it would be of interest to know whether SOCS-3 is also lower in the European farmers' environment despite a higher TLR2 expression.

In summary, *ex vivo* whole blood analysis of mRNA profiles in children infected with helminths compared to non-infected children living in a rural area in Ghana have shown that chronic helminth infections are associated with a lower expression of TLR2 and SOCS-3. The difference in the expression of SOCS-3 in helminth infected and uninfected children might result from the interaction of helminth derived molecules with the immune system leading to modulation of downstream signalling and induction of “modified” Th2 cells. Larger epidemiological studies will be needed to be able to test this hypothesis directly and to confirm that helminths modify the development of allergy by modulating the expression levels of TLR2 and SOCS-3.
